# Number of metastatic organs negatively affects the treatment sequence in patients with EGFR‐TKI failure

**DOI:** 10.1111/1759-7714.13360

**Published:** 2020-02-20

**Authors:** Takaaki Mizuno, Hidehito Horinouchi, Sho Watanabe, Jun Sato, Ryo Morita, Shuji Murakami, Yasushi Goto, Shintaro Kanda, Yutaka Fujiwara, Noboru Yamamoto, Yuichiro Ohe

**Affiliations:** ^1^ Department of Thoracic Oncology National Cancer Center Hospital Tokyo Japan; ^2^ Cancer Medicine The Jikei University Graduate School of Medicine Tokyo Japan

**Keywords:** Chemotherapy, epidermal growth factor receptor (*EGFR*), non‐small cell lung cancer, number of organs with metastasis, treatment sequence

## Abstract

**Background:**

Several studies have previously demonstrated the survival benefit of both EGFR‐TKI treatment and chemotherapy in patients with non‐small cell lung cancer (NSCLC) harboring *EGFR* mutations. The aim of the present study was to clarify the factors influencing the treatment sequence after failure of EGFR‐TKI therapy, focusing on the number of organs with metastasis (hereafter, metastatic organs).

**Methods:**

Between January 2010 and December 2016, consecutive patients with *EGFR*‐mutated NSCLC who were started on first‐line EGFR‐TKI were reviewed. The factors influencing withholding systemic chemotherapy and the post‐progression survival (PPS) after failure of EGFR‐TKI were investigated.

**Results:**

A total of 393 patients were started on first‐line EGFR‐TKI during the study period. After excluding patients maintained on EGFR‐TKI or who received osimertinib targeting secondary *EGFR* T790M, 297 patients were included in the analysis. Among these, 180 (60.6%) received chemotherapy after failure of EGFR‐TKI (TKI‐Ct group), while the remaining 117 (39.4%) received no chemotherapy (TKI‐only group). Multivariate analysis identified older age (≥75 years: odds ratio [OR] = 0.25, 95% confidence interval [CI]: 0.11–0.43, *P* < 0.001), poor performance status (PS) (≥2: OR = 0.06, 95% CI: 0.03–0.15, *P* < 0.001), and three or more metastatic organs (OR = 0.42, 95% CI: 0.22–0.80, *P* = 0.008) as being significantly associated with withholding of chemotherapy after failure of EGFR‐TKI.

**Conclusion:**

A relatively large number of metastatic organs and a poor PS were associated with the withholding of subsequent chemotherapy after failure of EGFR‐TKI in *EGFR*‐mutated NSCLC patients. Further research for patients with such a poor prognosis should be investigated in the future.

## Introduction

Epidermal growth factor receptor (*EGFR*) mutations as oncogenic driver mutations are encountered in 10% to 15% of non‐small‐cell lung carcinoma (NSCLC) patients in Western countries and approximately 50% of NSCLC patients in East‐Asian countries.^1^, ^2^, ^3^ EGFR‐tyrosine kinase inhibitor (EGFR‐TKI) monotherapy has been demonstrated to yield better disease control rates and survival outcomes than conventional chemotherapy in *EGFR*‐mutated NSCLC patients and has been the standard therapy for such patient population.^4^, ^5^, ^6^, ^7^ EGFR‐TKI therapy administered in combination with chemotherapy has been shown to yield a better prognosis than EGFR‐TKI therapy or chemotherapy alone.^8^, ^9^, ^10^ While 60% to 90% of the patients who receive chemotherapy as first‐line treatment receive subsequent EGFR‐TKI therapy, half of the patients administered first‐line EGFR‐TKI therapy fail to receive subsequent chemotherapy.^10^, ^11^, ^12^ Few studies have been conducted to examine the reasons why patients given first‐line EGFR‐TKI therapy often fail to receive subsequent chemotherapy.^11^, ^12^

In a recent study, Nakamura *et al*. reported that the number of metastatic organs is a prognostic factor affecting the survival after failure of first‐line EGFR‐TKI therapy.^13^ They suggested that a lower number of metastatic organs may reflect a higher degree of tumor shrinkage and a lower tumor burden and lead to a better prognosis.

Here, we hypothesized that the number of metastatic organs would also affect the treatment sequence in addition to the prognosis, and examined the factors that influence the withholding of subsequent chemotherapy after failure of first‐line EGFR‐TKI therapy, focusing on the number of metastatic organs.

## Methods

### Patient population

The data of consecutive patients who were started on first‐line EGFR‐TKI therapy between January 2010 and December 2016 at the National Cancer Center Hospital (Tokyo, Japan) were retrospectively reviewed. Patient characteristics after failure of EGFR‐TKI therapy were collected from the electronic medical records including the age, gender, Eastern Cooperative Oncology Group performance status (ECOG‐PS), *EGFR* status at diagnosis, response to first‐line EGFR‐TKI therapy according to the RECIST criteria (ver. 1.1), number of metastatic organs after failure of first‐line EGFR‐TKI therapy, and the main reason for withholding subsequent chemotherapy. Positive lymph nodes were counted collectively as one metastatic organ. Disease progression was defined as PD according to the RECIST criteria or symptomatic progression.

We divided the patients into two groups: the TKI‐chemotherapy (TKI‐Ct) group and the TKI‐only group. The TKI‐Ct group consisted of patients who had received chemotherapy (platinum doublet or single‐agent chemotherapy) after the failure of EGFR‐TKI therapy, while the TKI‐only group consisted of patients who did not receive any systemic treatment after the EGFR‐TKI therapy. This study was conducted with the approval of the institutional ethical review board (2015‐355).

### Systemic treatment

Patients with brain metastasis tended to receive erlotinib or afatinib treatment after local therapies such as whole‐brain radiotherapy or stereotactic radiotherapy for the brain metastasis. Patients without brain metastasis usually received gefitinib as the first‐line treatment. Follow‐up computed tomography for systemic lesions, including brain images, was performed every two to three months or when clinically indicated, to determine the disease status. After failure of EGFR‐TKI therapy (PD according to RECIST), some patients were continued on EGFR‐TKI therapy with the expectation of some clinical benefit. After discontinuation of the first‐line EGFR‐TKI therapy, many patients received systemic chemotherapy, including platinum‐containing regimens, docetaxel, S‐1 or immune checkpoint inhibitors.

### Statistical analysis

The purpose of this study was to identify the factors influencing the withholding of subsequent cytotoxic chemotherapies and the prognosis after failure of first‐line EGFR‐TKI therapy in *EGFR*‐mutated NSCLC patients. The post‐progression survival (PPS) was defined as the time from the documentation of disease progression after the start of first‐line EGFR‐TKI therapy to death from any cause. We also conducted a subgroup analysis to compare the PPS depending on the number of metastatic organs.

We used the *t*‐test or Mann‐Whitney U test to compare continuous variables and the chi‐square or Fisher's exact test to compare categorical variables to detect the differences between the groups. Spearman rank correlation coefficients were used to examine the association between pairs of variables and the correlation ≥0.2 was defined as a meaningful correlation. The estimated survival was calculated using the Kaplan‐Meier method, with determination of the 95% confidence intervals (CIs) and comparisons between the groups performed by the log‐rank test. To detect the independent prognostic factors for determination of the treatment sequence and survival prognosis, a logistic regression model and Cox proportional hazards model were applied. All analyses were performed using the Statistical Package for the Social Sciences (SPSS v.21; SPSS, Inc., Chicago, IL, USA). Two‐sided *P* < 0.05 was considered to indicate a statistically significant difference.

## Results

### Patient demographics

**Figure 1:**
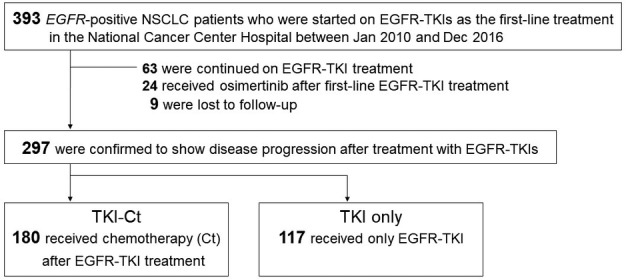
Patient selection. EGFR, epidermal growth factor receptor; NSCLC, non‐small cell lung cancer; TKI, tyrosine kinase inhibitor.

In total, 393 *EGFR*‐positive NSCLC patients were started on first‐line EGFR‐TKI therapy during the study period (Fig 1). At the data cutoff (30 June 2018), 330 patients experienced disease progression with first‐line EGFR‐TKI. A total of 265 patients progressed before approval of osimertinib in Japan (March 2016), while 67 patients progressed after approval of osimertinib. After excluding patients who were continued on the first‐line EGFR‐TKI regimen or received osimertinib as a drug targeting T790M (related to the development of acquired resistance to first‐line EGFR‐TKI treatment), 297 patients remained. Among these, 180 (60.6%) received chemotherapy after failure of EGFR‐TKI therapy (TKI‐Ct group), while the remaining 117 (39.4%) received no chemotherapy (TKI‐only group). By the time of censoring of the data, 181 patients (60.9%) had died. The patient demographic characteristics are summarized in Table 1. The median age, ECOG‐PS, *EGFR* mutation status and number of metastatic organs were significantly different between the two groups. Continuing EGFR‐TKI beyond progression was seen in 58 (32.2%) in the TKI‐ct group and 53 (45.3%) in the TKI only group (*P* = 0.023). Among the TKI‐ct group, subsequent platinum‐based doublet chemotherapy was administered in 137 patients and single‐agent chemotherapy in 43 patients.

**Table 1:** Patient characteristics after failure of first‐line EGFR‐TKI treatment

	TKI‐Ct (*n* = 180)	TKI only (*n* = 117)	*P*‐value
Mean age, years	64.2	70.1	<0.001
<75 years, *n* (%)	152 (84.4)	67 (57.3)	—
≥75 years, *n* (%)	28 (15.6)	50 (42.7)	—
Female, *n* (%)	111 (61.7)	82 (70.1)	0.261
ECOG‐PS, *n* (%)	—	—	0.001
0–1	170 (94.4)	54 (46.2)	—
2–4	10 (5.6)	46 (39.3)	—
NE	0 (0.0)	17 (14.5)	—
Histology	—	—	0.059
Adenocarcinoma	179	112	—
Squamous cell carcinoma	0	3	—
Adenosquamous carcinoma	1	2	—
*EGFR* status, *n* (%)	—	—	0.028
Exon 19 deletion	105 (58.3)	50 (42.7)	—
L858R	69 (38.3)	63 (53.8)	—
Other	6 (3.3)	4 (3.4)	—
Stage	—	—	0.054
III/IV	120	65	—
Recurrence	60	52	—
First‐line EGFR‐TKI regimen used, *n* (%)	—	—	0.216
Gefitinib	149 (82.8)	90 (76.9)	—
Erlotinib	8 (4.4)	9 (7.7)	—
Afatinib	23 (12.8)	18 (15.3)	—
Response to first‐line EGFR‐TKI treatment, n (%)	—	—	0.210
CR or PR	113 (62.8)	65 (55.6)	—
SD or PD	64 (35.6)	50 (42.7)	—
NE	3 (1.7)	2 (1.7)	—
CNS metastases, n (%)	—	—	<0.001
Present	28 (15.6)	50 (42.7)	—
Absent	152 (84.4)	67 (57.3)	—
Median number of organs with metastasis, (range)	2 (0–8)	2 (0–6)	0.259
Number of organs with metastasis, n (%)	—	—	0.012
≤2	123 (68.3)	61 (52.1)	—
≥3	53 (29.4)	50 (42.7)	—
NE	4 (2.2)	6 (5.1)	—

CNS, central nervous system; CR, complete response; Ct, chemotherapy; ECOG, Eastern Cooperative Oncology Group; EGFR, epidermal growth factor receptor; NE, not evaluated; PD, progressive disease; PR, partial response; PS, performance status; SD, stable disease; TKI, tyrosine kinase inhibitor.

### Reasons for withholding subsequent chemotherapy

The causes of withholding of subsequent chemotherapy after failure of EGFR‐TKI therapy are shown in Table 2. The most frequent reason was PS deterioration, mainly because of the presence of leptomeningitis or brain metastases, followed by older age, patient preference, and systemic progression without local symptoms. Approximately one half of the patients could not receive chemotherapy because of cancer‐related regional complications, such as metastases in the central nervous system (CNS), pleura or bone.

**Table 2:** Causes for failure to receive subsequent chemotherapy

Cancer‐related (*n* = 72)	*n*	Noncancer related (*n* = 45)	*n*
PS deterioration	50	Older age	25
Leptomeningitis	27	Patients' preference	14
Brain metastases	11	Comorbidities	6
Malignant pleural effusion	5	—	—
Carcinomatous pericarditis	4	—	—
Bone metastasis	3	—	—
Systemic progression without local symptoms	12	—	—
Adverse events during EGFR‐TKI treatment	3	—	—
Other cancer‐related complications	7	—	—

EGFR, epidermal growth factor receptor; PS, performance status; TKI, tyrosine kinase inhibitor.

We conducted univariate and multivariate logistic analyses to investigate the factors associated with withholding of chemotherapy after failure of EGFR‐TKI treatment. A multivariate analysis with candidate prognostic factors in univariate analysis with *P*‐value less than 0.05, identified older age (75 years or more: odds ratio (OR) = 0.21, 95% CI: 0.11–0.43, *P* = <0.001), poor ECOG‐PS (two or more: OR = 0.06, 95% CI: 0.03–0.15, *P* < 0.001), and ≥3 metastatic organs (OR = 0.42, 95% CI: 0.22–0.80, *P* = 0.008) as being significantly associated with the withholding of chemotherapy after failure of first‐line EGFR‐TKI therapy (Table 3).

**Table 3:** Factors associated with the administration of chemotherapy after EGFR‐TKI treatment

	Univariate			Multivariate		
Factor	OR	95% CI	*P* value	OR	95% CI	*P* value
Age in years (≥75 vs. <75)	0.25	0.14–0.43	<0.001	0.21	0.11–0.43	<0.001
Gender (male vs. female)	1.52	0.92–2.49	0.101	—	—	—
*EGFR* status (del 19 vs. L858R)	1.98	1.23–3.19	0.005	1.58	0.85–2.93	0.148
Smoking status (ever smoker vs. never smoker)	1.65	1.01–2.69	0.045	0.98	0.52–1.87	0.958
Best response to first‐line TKI (CR/PR vs. SD/PD)	0.66	0.41–1.07	0.091	—	—	—
ECOG‐PS (≥2 vs. ≤1)	0.04	0.18–0.81	<0.001	0.06	0.03–0.15	<0.001
Number of organs with metastasis (≥3 vs. ≤2)	0.53	0.33–0.85	0.009	0.42	0.22–0.80	0.008

CI, confidence interval; CR, complete response; ECOG, Eastern Cooperative Oncology Group; EGFR, epidermal growth factor receptor; OR, odds ratio; PD, progressive disease; PR, partial response; PS, performance status; SD, stable disease; TKI, tyrosine kinase inhibitor.

### Survival analysis

We also conducted a multivariate analysis to investigate the factors associated with the survival after failure of EGFR‐TKI therapy, which identified a poor PS (≥2: hazard ratio (HR) = 3.90, 95% CI 2.63–5.78, *P* < 0.001) and ≥3 metastatic organs (HR = 2.55, 95% CI 1.85–3.50, *P* < 0.001) as being independent prognostic factors after failure of EGFR‐TKI therapy (Table 4).

**Table 4:** Factors associated with post‐progression survival after failure of EGFR‐TKI

	Univariate			Multivariate		
Factor	HR	95% CI	*P*‐value	HR	95% CI	*P*‐value
Age in years (≥75 vs. <75)	1.28	0.91–1.80	0.162	—	—	—
Gender (male vs. female)	0.89	0.65–1.21	0.445	—	—	—
*EGFR* status (del 19 vs. L858R)	0.73	0.54–0.98	0.037	0.89	0.65–1.22	0.461
Smoking status (ever smoker vs. never smoker)	0.92	0.68–1.24	0.574	—	—	—
Best response to first‐line TKI (CR/PR vs. SD/PD)	1.20	0.89–1.62	0.091	—	—	—
ECOG‐PS (≥2 vs. ≤1)	4.53	3.13–6.56	<0.001	3.90	2.63–5.78	<0.001
Number of organs with metastasis (≥3 vs. ≤2)	2.33	1.73–3.15	<0.001	2.55	1.85–3.50	<0.001

CI, confidence interval; CNS, central nervous system; CR, complete response; ECOG, Eastern Cooperative Oncology Group; EGFR, epidermal growth factor receptor; HR, hazard ratio; PD, progressive disease; PR, partial response; PS, performance status; SD, stable disease; TKI, tyrosine kinase receptor inhibitor.

**Figure 2:**
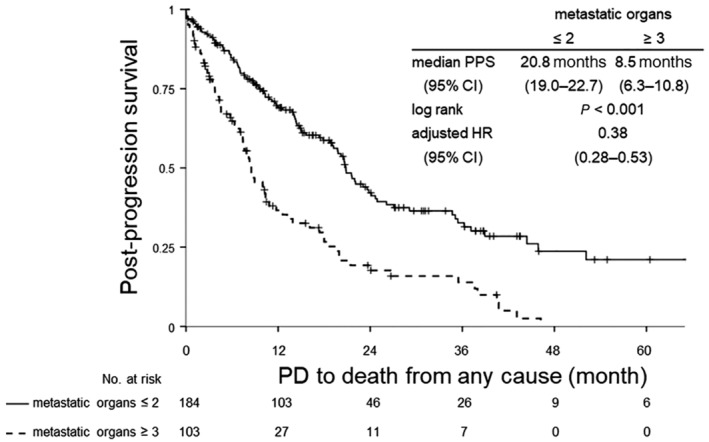
Kaplan‐Meier survival analysis of PPS, from documentation of disease progression after EGFR‐TKI therapy to death from any cause in the overall population. CI, confidence interval; EGFR, epidermal growth factor receptor; HR, hazard ratio; PD, progression disease; PPS, post‐progression survival; TKI, tyrosine kinase inhibitor.

**Figure 3:**
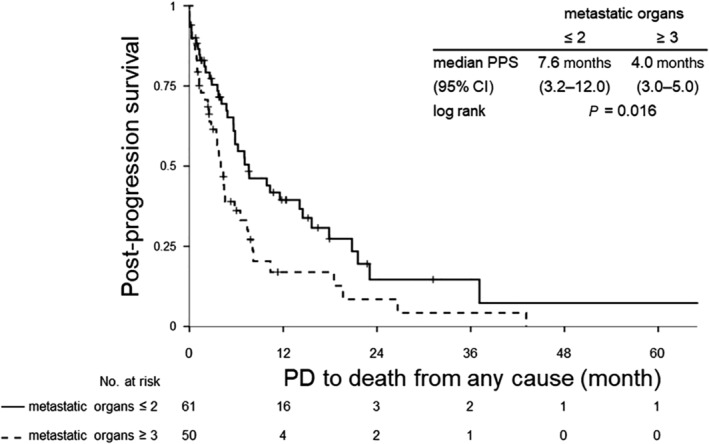
Kaplan‐Meier survival analysis of PPS, from documentation of disease progression after EGFR‐TKI therapy to death from any cause in patients who received only EGFR‐TKI treatment as their systemic treatment. CI, confidence interval; EGFR, epidermal growth factor receptor; HR, hazard ratio; PD, progression disease; PPS, post‐progression survival; TKI, tyrosine kinase inhibitor.

**Figure 4:**
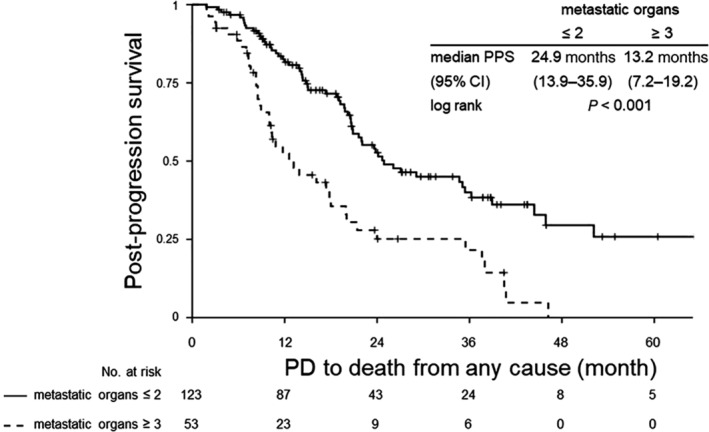
Kaplan‐Meier survival analysis of PPS, from documentation of disease progression after EGFR‐TKI therapy to death from any cause in patients who received chemotherapy after the failure of EGFR‐TKI therapy. CI, confidence interval; EGFR, epidermal growth factor receptor; HR, hazard ratio; PD, progression disease; PPS, post‐progression survival; TKI, tyrosine kinase inhibitor.

Figures 2, 3, 4 shows the Kaplan‐Meier curves for PPS after failure of EGFR‐TKI therapy until death from any cause in the overall study population (Fig 2), in patients who did not receive subsequent chemotherapy (Fig 3) and in patients who received subsequent chemotherapy (Fig 4). The number of metastatic organs at the time of documentation of disease progression after EGFR‐TKI therapy was significantly related to survival, irrespective of whether the patients received subsequent chemotherapy or not, in each of the patient populations. In the entire population, patients with a larger number of metastatic organs showed a worse prognosis with a HR of 0.38 (95% CI: 0.28–0.53) after adjustments for differences in the patient characteristics and a significant difference in the median PPS depending on the number of metastatic organs (20.8 months in patients with ≤2 metastatic organs (95% CI: 19.0–22.7) and 8.5 months in patients with ≥3 metastatic organs (95% CI: 6.3–10.8) (Fig 2). Patients who did not receive subsequent chemotherapy showed disappointing survival outcomes in each of the arms (Fig 3). On the other hand, patients who received subsequent chemotherapy showed relatively better prognosis; in particular, patients with ≤2 metastatic organs arm showed a median PPS of more than two years, even after failure of first‐line EGFR‐TKI therapy (Fig 4).

## Discussion

This study is the largest study until date conducted to examine the factors influencing the withholding of subsequent cytotoxic chemotherapy after failure of treatment with a first‐ or second‐generation EGFR‐TKI in consecutive *EGFR*‐mutated NSCLC patients. We identified the number of metastatic organs and PS as independent factors associated with withholding of subsequent chemotherapy and a poor prognosis.

EGFR‐TKI therapy has improved the survival and quality of life outcomes for NSCLC patients harboring *EGFR* mutations.^4^, ^5^, ^6^, ^7^, ^8^, ^9^, ^10^, ^14^, ^15^ Although several studies have shown that chemotherapy has an important role in improving the prognosis in *EGFR*‐mutated NSCLC patients,^8^, ^9^, ^10^ a proportion of patients miss the opportunity to receive subsequent chemotherapy.^10^, ^11^, ^12^ In the present study, the initiation of subsequent chemotherapy after EGFR‐TKI therapy contributed to a better prognosis, regardless of the number of metastatic organs. In particular, patients with a fewer number of metastatic organs after failure of EGFR‐TKI therapy showed a median PPS of more than two years, despite the failure of EGFR‐TKI therapy. This suggests that appropriate use of chemotherapy might yield a good prognosis in selected patients, and that the decrease in the number of metastatic organs after the initial therapy may contribute to the better prognosis, as shown by Nakamura *et al*.^13^ Recently, one randomized phase II study conducted to evaluate the efficacy of local ablative therapy (LAT) for patients with a controlled primary solid tumor and one to five metastatic lesions was reported.^16^ This study showed a better survival after LAT for oligometastatic disease as compared to the standard of care, regardless of the increase of severe adverse events. LAT would also be a potential option for patients with a larger number of metastases if all the lesions were irradiable in parenchymal organs.

Kawaguchi *et al*. reported that the PS and patient preference could influence the treatment sequence in patients receiving first‐line EGFR‐TKI therapy. They also reviewed the initial recurrence site after failure of EGFR‐TKI therapy and found CNS as the most frequent extrathoracic metastatic site. However, what causes the deterioration of the PS remains unknown. Therefore, we conducted an evaluation to understand, in detail, why patients could not make the transition to chemotherapy after failure of EGFR‐TKI therapy and elucidated the cause; the analysis identified PS deterioration because of CNS metastasis, which mostly manifests as leptomeningitis, as the most frequent cause. As NSCLC patients with leptomeningitis or brain metastasis may also benefit from chemotherapy,^17^, ^18^, ^19^ close surveillance for the detection of CNS metastasis and a multidisciplinary approach for the CNS metastases may contribute to a better treatment sequence and prognosis.

Our study had several limitations. First, this was a retrospective study conducted at a single institution and the selection among the three first‐line EGFR‐TKI treatment agents available and the timing of change of treatment were left to the discretion of the attending physician and the patients' preference. Although heterogeneous, 47% to 62% of patients in each EGFR‐TKI treatment group received subsequent chemotherapy and the differences were not significant. Therefore, these patient cohorts could be considered as relatively uniform. Second, we excluded patients who were continued on the first‐line EGFR‐TKI treatment or received osimertinib; therefore, long responders tended to be excluded from the study and the PPS could have been underestimated. The present study focused on first‐ or second‐generation EGFR‐TKIs such as gefitinib, erlotinib or afatinib; therefore, further investigation is warranted to verify if the same results can be replicated for the third‐generation EGFR‐TKIs, for example, osimertinib. Third, the present study included patients treated with first‐ or second‐generation EGFR‐TKIs, so we should consider the T790M associated acquired resistance and subsequent osimertinib treatment. Because there was only 17% (67/393) of our study population who progressed after approval of osimertinib in Japan, the influence can be considered minimal. Fourth, there were differences in the patient background characteristics, including age, ECOG‐PS, *EGFR* mutation status, and number of metastatic organs, between the groups. We adjusted this intersubgroup heterogeneity using multivariate analyses and identified factors which significantly affected the outcomes.

In conclusion, a larger number of metastatic organs and PS deterioration are important factors for withholding subsequent chemotherapy after failure of EGFR‐TKI therapy and poor prognosis in *EGFR*‐mutated NSCLC patients. Particular attention should be given to the treatment of such patients with a poor prognosis and further studies should be carried out in the future.

## Disclosure

H.H. reports personal fees from Chugai, Astra Zeneca. S.M. reports personal fees from Astra Zeneca, Chugai, Boehringer Ingelheim, Y.G. reports personal fees from Chugai, Boehringer Ingelheim, Pfizer, Astra Zeneca. S.K. reports personal fees from Astra Zeneca, Chugai. Y.F. reports personal fees from Astra Zeneca. NY reports personal fees from Chugai, Astra Zeneca, Pfizer, Boehringer Ingelheim. Y.O. reports personal fees from Pfizer, Astra Zeneca, Chugai, Boehringer Ingelheim. H.H. reports grant from Chugai, Astra Zeneca. S.K. reports grants from Astra Zeneca. Y.F. reports grants from Astra Zeneca, Chugai. N.Y. reports grants from Chugai, Pfizer, Boehringer Ingelheim. Y.O. reports grants from Pfizer, Astra Zeneca, Chugai.

The authors report no conflicts of interest.

## References

[1] Rosell R , Moran T , Queralt C *et al* Screening for epidermal growth factor receptor mutations in lung cancer. N Engl J Med 2009; 361: 958–67. 19692684 10.1056/NEJMoa0904554

[2] Scoccianti C , Vesin A , Martel G *et al* Prognostic value of TP53, KRAS and EGFR mutations in nonsmall cell lung cancer: The EUELC cohort. Eur Respir J 2012; 40: 177–84. 22267755 10.1183/09031936.00097311

[3] Peters S , Adjei AA , Gridelli C *et al* Metastatic non‐small‐cell lung cancer (NSCLC): ESMO Clinical Practice Guidelines for diagnosis, treatment and follow‐up. Ann Oncol 2012; 23 (Suppl 7): vii56–64. 22997455 10.1093/annonc/mds226

[4] Mok TS , Wu YL , Thongprasert S *et al* Gefitinib or carboplatin‐paclitaxel in pulmonary adenocarcinoma. N Engl J Med 2009; 361: 947–57. 19692680 10.1056/NEJMoa0810699

[5] Mitsudomi T , Morita S , Yatabe Y *et al* Gefitinib versus cisplatin plus docetaxel in patients with non‐small‐cell lung cancer harbouring mutations of the epidermal growth factor receptor (WJTOG3405): An open label, randomised phase 3 trial. Lancet Oncol 2010; 11: 121–8. 20022809 10.1016/S1470-2045(09)70364-X

[6] Maemondo M , Inoue A , Kobayashi K *et al* Gefitinib or chemotherapy for non‐small‐cell lung cancer with mutated EGFR. N Engl J Med 2010; 362: 2380–8. 20573926 10.1056/NEJMoa0909530

[7] Sequist LV , Yang JC‐H , Yamamoto N *et al* Phase III study of afatinib or cisplatin plus pemetrexed in patients with metastatic lung adenocarcinoma with EGFR mutations. J Clin Oncol 2013; 31: 3327–34. 23816960 10.1200/JCO.2012.44.2806

[8] Inoue A , Kobayashi K , Maemondo M *et al* Updated overall survival results from a randomized phase III trial comparing gefitinib with carboplatin‐paclitaxel for chemo‐naive non‐small cell lung cancer with sensitive EGFR gene mutations (NEJ002). Ann Oncol 2013; 24: 54–9. 22967997 10.1093/annonc/mds214

[9] Okamoto I , Morita S , Tashiro N *et al* Real world treatment and outcomes in EGFR mutation‐positive non‐small cell lung cancer: Long‐term follow‐up of a large patient cohort. Lung Cancer 2018; 117: 14–9. 29496250 10.1016/j.lungcan.2018.01.005

[10] Zhou C , Wu YL , Chen G *et al* Final overall survival results from a randomised, phase III study of erlotinib versus chemotherapy as first‐line treatment of EGFR mutation‐positive advanced non‐small‐cell lung cancer (OPTIMAL, CTONG‐0802). Ann Oncol 2015; 26: 1877–83. 26141208 10.1093/annonc/mdv276

[11] Kato Y , Hotta K , Takigawa N *et al* Factor associated with failure to administer subsequent treatment after progression in the first‐line chemotherapy in EGFR‐mutant non‐small cell lung cancer: Okayama Lung Cancer Study Group experience. Cancer Chemother Pharmacol 2014; 73: 943–50. 24633759 10.1007/s00280-014-2425-9

[12] Kawaguchi Y , Okano T , Kakihana M , Kajiwara N , Ohira T , Ikeda N . Transition rate from EGFR‐TKI to cytotoxic chemotherapy patients with EGFR mutation‐positive lung adenocarcinoma. Anticancer Res 2018; 38: 3127–32. 29715152 10.21873/anticanres.12574

[13] Nakamura A , Inoue A , Morita S *et al* Phase III study comparing gefitinib monotherapy (G) to combination therapy with gefitinib, carboplatin, and pemetrexed (GCP) for untreated patients (pts) with advanced non‐small cell lung cancer (NSCLC) with EGFR mutations (NEJ009). J Clin Oncol 2018; 36: 9005–5.

[14] Oizumi S , Kobayashi K , Inoue A *et al* Quality of life with gefitinib in patients with EGFR‐mutated non‐small cell lung cancer: Quality of life analysis of North East Japan Study Group 002 Trial. Oncologist 2012; 17: 863–70. 22581822 10.1634/theoncologist.2011-0426 PMC3380886

[15] Rosell R , Carcereny E , Gervais R *et al* Erlotinib versus standard chemotherapy as first‐line treatment for European patients with advanced EGFR mutation‐positive non‐small‐cell lung cancer (EURTAC): A multicentre, open‐label, randomised phase 3 trial. Lancet Oncol 2012; 13: 239–46. 22285168 10.1016/S1470-2045(11)70393-X

[16] Palma DA , Olson R , Harrow S *et al* Stereotactic ablative radiotherapy versus standard of care palliative treatment in patients with oligometastatic cancers (SABR‐COMET): A randomised, phase 2, open‐label trial. Lancet 2019; 393: 2051–8. 30982687 10.1016/S0140-6736(18)32487-5

[17] Park JH , Kim YJ , Lee JO *et al* Clinical outcomes of leptomeningeal metastasis in patients with non‐small cell lung cancer in the modern chemotherapy era. Lung Cancer 2012; 76: 387–92. 22186628 10.1016/j.lungcan.2011.11.022

[18] Liao BC , Lee JH , Lin CC *et al* Epidermal growth factor receptor tyrosine kinase inhibitors for non‐small‐cell lung cancer patients with leptomeningeal carcinomatosis. J Thorac Oncol 2015; 10: 1754–61. 26334749 10.1097/JTO.0000000000000669

[19] Lee DH , Han JY , Kim HT *et al* Primary chemotherapy for newly diagnosed nonsmall cell lung cancer patients with synchronous brain metastases compared with whole‐brain radiotherapy administered first: Result of a randomized pilot study. Cancer 2008; 113: 143–9. 18459180 10.1002/cncr.23526

